# The intestine: A highly dynamic microenvironment for IgA plasma cells

**DOI:** 10.3389/fimmu.2023.1114348

**Published:** 2023-02-16

**Authors:** Katharina Pracht, Jens Wittner, Fritz Kagerer, Hans-Martin Jäck, Wolfgang Schuh

**Affiliations:** Division of Molecular Immunology, Department of Internal Medicine 3, Nikolaus-Fiebiger-Center, University Hospital Erlangen, Friedrich-Alexander-Universität Erlangen-Nürnberg, Erlangen, Germany

**Keywords:** IgA, IgA plasma cells, intestinal epithelial cell, survival niche, intestinal epithelial barrier, Aryl hydrocarbon (Ah) receptor, plasma cell

## Abstract

To achieve longevity, IgA plasma cells require a sophisticated anatomical microenvironment that provides cytokines, cell-cell contacts, and nutrients as well as metabolites. The intestinal epithelium harbors cells with distinct functions and represents an important defense line. Anti-microbial peptide-producing paneth cells, mucus-secreting goblet cells and antigen-transporting microfold (M) cells cooperate to build a protective barrier against pathogens. In addition, intestinal epithelial cells are instrumental in the transcytosis of IgA to the gut lumen, and support plasma cell survival by producing the cytokines APRIL and BAFF. Moreover, nutrients are sensed through specialized receptors such as the aryl hydrocarbon receptor (AhR) by both, intestinal epithelial cells and immune cells. However, the intestinal epithelium is highly dynamic with a high cellular turn-over rate and exposure to changing microbiota and nutritional factors. In this review, we discuss the spatial interplay of the intestinal epithelium with plasma cells and its potential contribution to IgA plasma cell generation, homing, and longevity. Moreover, we describe the impact of nutritional AhR ligands on intestinal epithelial cell-IgA plasma cell interaction. Finally, we introduce spatial transcriptomics as a new technology to address open questions in intestinal IgA plasma cell biology.

## The structure of the intestinal epithelium

The intestine consists of the small and the large intestine. The small intestine starts at the pylorus and is subdivided into three main parts: the duodenum, the jejunum, and the ileum. The large intestine consists of the caecum, the proximal colon, the transverse colon, the distal colon, the rectum, and ends at the anus. The cellular composition of the epithelium as well as that of the lamina propria (LP) differs along the segments of the intestine, concomitant with the different physiological functions and the different bacterial densities of the small intestine and the colon. The primary function of the small intestine is digestion and the absorption of nutrients. To increase the surface for food absorption, the small intestine is characterized by the presence of villi, whereas villi cannot be found in the caecum and the colon. The main function of the colon is water reabsorption and removal of undigested food. The colon contains the highest density of commensal bacteria (1, 2). Despite their anatomical and functional differences, all segments of the intestine are lined by the mono-layered intestinal epithelium. The intestinal epithelial cells (IECs) and the immune cell composition differs along the segments of the intestine, concurrent with the different physiological functions and the bacterial load of the small intestine and the colon.

**Figure 1:**
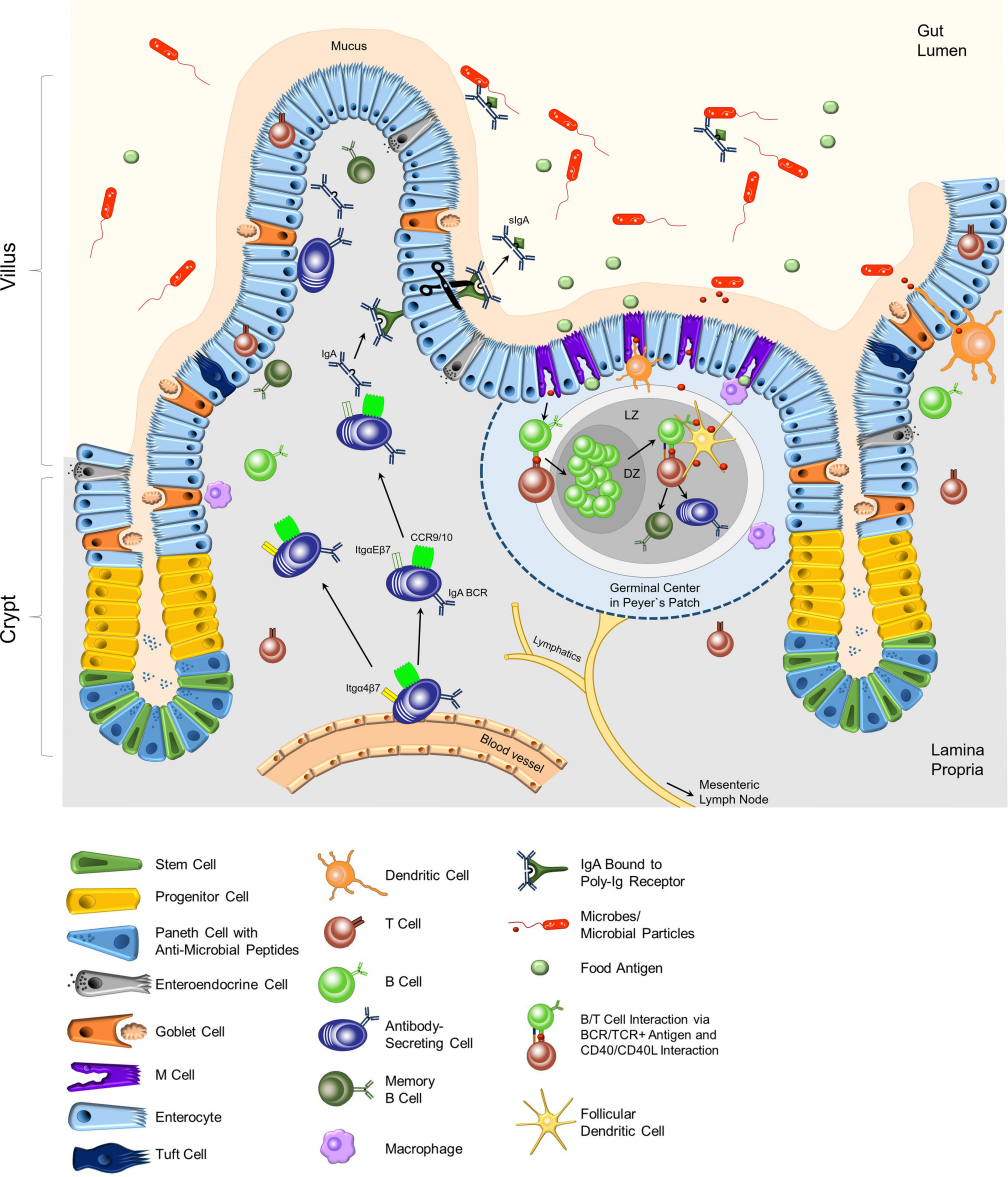
Composition of the epithelium and the lamina propria of the small intestine. In the crypt region, paneth cells secrete anti-microbial peptides into the mucus. Stem cells differentiate into the specialized cells of the intestinal epithelial layer in the villi. Goblet cells secrete mucus, which keeps microbes at bay. Immune cells, mostly T cells, can be located as intra-epithelial lymphocytes (IEL). M cells are located adjacent to Peyer`s patches (PPs), which are part of the lymphoid organs of the intestinal tract. M cells transport food antigens or microbial particles to DCs, macrophages, and B and T lymphocytes in the PPs. Antigen-specific B and T lymphocytes induce a germinal center with the dark zone (DZ) and light zone (LZ) in the PPs. Here, activated B cells undergo affinity maturation and class switch recombination with the help of T cells and follicular dendritic cells, resulting in mainly IgA-class-switched, antigen-specific antibody-secreting cells and memory B cells. Dendritic cells support the activation of adaptive immune cells by acquiring antigens through the epithelial layer and transporting them to the PPs or the mLNs. IgA-secreting cells migrate from the blood vessels to the epithelial layer, attracted by cytokine gradients (CCR9/10) and guided by integrins. Dimeric IgA that binds to p-Ig receptors on epithelial cells is transported through the epithelium. Secreted IgA (sIgA) in the lumen binds to specific antigens and regulates the intestinal microbiota composition. BCR, B cell receptor; Itg, integrin.

Mucosal surfaces are the most critical entry sites for pathogens into our body. Therefore, a sophisticated mucosal defense system evolved that combines chemical, physical, and cellular barriers. The mucosal immune system in the intestine consists of immune cells and the intestinal epithelium that orchestrates innate as well as adaptive immune responses. The epithelium constitutes the interface between the gut lumen and the LP. Its functions include the uptake of nutrients and antigens on the one hand, as well as microbial sensing and exclusion of pathogens on the other hand. The intestinal epithelium consists of an epithelial cell monolayer, the LP and the muscularis mucosae. It can be subdivided into the crypt area where stem cells are located and the villus area (**Figure 1**). IECs are connected by tight junctions and are attached to a basement membrane that consists of laminin, collagen, fibronectin and other extracellular matrix (ECM) components. The basement membrane provides a platform for cell adhesion, migration, differentiation, and functions as a barrier (3, 4). In addition, it harbors pores of various sizes that allow immune cells (e.g., intra-epithelial T cells) to physically interact with epithelial cells (4, 5).

IECs originate from Lgr5^+^ stem cells in the crypts and differentiate into specialized epithelial cells (6). Enterocytes represent the majority of IECs and their primary function is the absorption of nutrients. Besides enterocytes, the epithelium contains specialized tuft cells, enteroendocrine cells, goblet cells, paneth cells, and microfold (M) cells (**Figure 1**). The intestinal epithelium is characterized by a high turn-over with an average turn-over time of 4-5 days. Stem cells in the crypts constantly divide and give rise to so-called transit-amplifying cells or progenitor cells (6–8). These cells further differentiate into specialized IECs. During this process, newly formed cells move from the crypt towards the villus tip. IECs that reach the villus tip undergo cell death, and are shed off and replaced. The proliferation, differentiation, and cell death processes of IECs are regulated by gradients of ligands of the Wnt, the BMP, the Notch, and the ephrin signaling pathways (6–8). The following paragraphs will briefly describe the various cell epithelial types and their biological functions (**Figure 1**).

Tuft cells are chemosensory cells characterized by their unique bottle-shaped morphology with brush-like apical microvilli. Tuft cells express taste receptors on their surface, such as TRPM-5. They utilize components of the “taste receptor” signaling cascades, and are the source of IL-25, a cytokine that acts on innate lymphoid cells (ILC) 2 and natural killer T (NKT) cells, and contributes to anti-helminth immune responses (9). Enteroendocrine cells (EECs) produce a multitude of hormones, neurotransmitters and neuropeptides that in turn regulate gut motility, digestion, food absorption, and insulin secretion. The function of EECs is modulated by nutrients and microbiota metabolites, such as short-chain fatty acids (SCFAs) (10). Mucin secretion by goblet cells is the source of mucus production (11, 12). Mucins are glycoproteins consisting of a core protein and O-linked glycans and can be subdivided in gel-forming mucins and transmembrane mucins. Gel-forming mucins are characterized by homo-dimerization and the formation of networks. The predominant component of mucus in the intestine is the gel-forming mucin Muc2. The biological relevance of Muc2 was demonstrated in Muc2-knockout mice which develop adenocarcinomas and colorectal cancer (13). The frequencies of goblet cells increase from the small intestine to the colon, where approximately 25% of all IECs are goblet cells. Consequently, the mucus thickness differs between the small intestine and the colon, with a thickening of the mucus in the colon (14). Paneth cells are localized in the crypts of the small intestine but are absent in the colon. Their biological function is the secretion of anti-microbial peptides (AMP) to the gut lumen. AMPs include, amongst others, defensins, lysozyme, secretory phospholipase A2, and RegIII (15, 16). Defensins can insert into the bacterial membranes where they form pores and thereby, disrupt the membrane or metabolic processes (16). RegIII proteins bind peptidoglycans on gram-positive bacteria and damage their cell wall (17).

## The effect of the intestinal epithelium and microenvironment on IgA plasma cell generation

The intestinal epithelium constitutes the interface between the gut lumen which contains bacteria, their metabolites, nutrients as well as food antigens on one side, and the LP containing immune cells on the other side. IECs together with intra-epithelial lymphocytes (IELs) and immune cells within the LP are involved in sensing and transporting antigens to the Peyer`s patches (PPs) and the mesenteric lymph nodes (mLNs) and maintaining epithelial integrity (18). The majority of IELs are specialized T cells that are localized within the epithelium. IELs express C-C chemokine receptor (CCR) 9 as well as integrin αE (CD103) chain in combination with the integrin β7 chain on their cell surface (19). Integrin αEβ7 mediates the binding to E-Cadherin and contributes to the retention of IELs in the epithelium. IELs function as sentinels and support the homeostasis of the epithelium and its integrity. Moreover, CD103^+^ dendritic cells (DCs) are recruited to the epithelium and are instrumental in antigen sampling by forming protrusions through the epithelium to capture antigen from the gut lumen. Antigens are subsequently processed, and peptide fragments derived from the antigen are presented on MHC II molecules on the cell surface. Antigen-presenting DCs migrate to the PPs and mLNs to activate antigen-specific T cells (20).

Antigen transport to the LP is also mediated by M cells in collaboration with DCs as well as Macrophages that closely interact with them. M cells are located in so-called follicle-associated epithelium adjacent to the PPs and isolated lymphocyte follicles (ILFs) (21). M cells are instrumental in luminal antigen sampling and transport. Their specific structure enables the close contact with DCs and macrophages which take up and process antigens, and subsequently present antigen peptide fragments of the antigen on their MHC II molecules. Antigen-presenting CD103^+^ DCs migrate to the PP or mLNs to prime antigen-specific T cells. PPs as well as mLNs are secondary lymphoid organs and are structurally divided into a B cell and a T cell zone. B cells residing in PPs and mLNs bind antigen *via* their B cell receptor (BCR) and are activated upon cognate interaction with antigen-specific T cells. As a result, activated B cells proliferate, undergo class switch recombination (CSR) to Immunoglobulin (Ig)A and somatic hypermutation (SHM) induced by Activation-induced cytidine deaminase (AID) within the germinal center (GC) reaction in PPs and mLNs (**Figure 1**) (22–24). IgA plasma cells and IgA memory B cells derive from activated B cells in the GC reaction. Furthermore, IgA plasma cell generation also occurs in isolated lymphoid follicles (ILFs) in the LP (25, 26). Class switch to IgA is triggered by the cytokine transforming growth factor-β (TGF-β) in cooperation with the vitamin A metabolite retinoic acid (RA). TGF-β is produced by various cell types, including regulatory T cells (Tregs), follicular T helper (T_FH_) cells, DCs, eosinophils, and also B cells (23, 27–29). Furthermore, DCs located in the PPs and mLNs express the enzymes ALDH1 and ALDH2 that are involved in RA generation (29, 30). Moreover, IL-21 provided by T_FH_ cells enhances the CSR to IgA (27). Besides T cell-dependent IgA class switch, T cell-independent CSR to IgA has also been described (22, 31). Grasset and colleagues demonstrated that signals triggered by the transmembrane activator and CAML interactor (TACI) receptor on B cells induce CSR to IgA in the absence of T cells (32).

Microbial sensing by IECs is mediated by multiple surface and intracellular Toll-like receptors (TLR) (33). In response to microbial sensing through TLRs, IECs promote the homeostasis of the epithelium and its integrity by promoting cell survival and repair. Furthermore, the microbiota – IEC interplay regulates mucus and AMP-secretion. Moreover, upon TLR activation, IECs produce crucial cytokines and chemokines that orchestrate immune responses, such as chemokine (C-C motif) ligand (CCL) 25, CCL28, a proliferation-inducing ligand (APRIL), B cell-activating factor (BAFF), IL-25, RA, TGF-β as well as thymic stromal lymphopoietin (TSLP) (34, 35). Furthermore, the cytokines TSLP, TGF-β and RA induce DCs and macrophages to provide tolerogenic signals, such as the secretion of IL-10 (36, 37). Homing of immune cells is promoted by TGF-β and RA as both cytokines were shown to be implicated in the upregulation of integrins β7 and αE on immune cells. Moreover, RA was also shown to upregulate the gut-homing receptor CCR9 on T and B cells (38, 39). In addition, TGF-β induces integrin αE expression in T cells, especially in CD8^+^ tissue-resident T cells (TRM) (40–45). Moreover, TGF-β, RA, and nitric-oxid (NO) produced by IECs are key cytokines for the induction of IgA CSR of B cells (39, 46–54). Importantly, BAFF and APRIL produced by IECs support B cell and plasma cell survival, respectively (55–57). In this context, IEC-derived TSLP also fosters additional APRIL and BAFF production by DCs (58).

## Homing of IgA plasmablasts to the intestinal lamina propria

**Figure 2:**
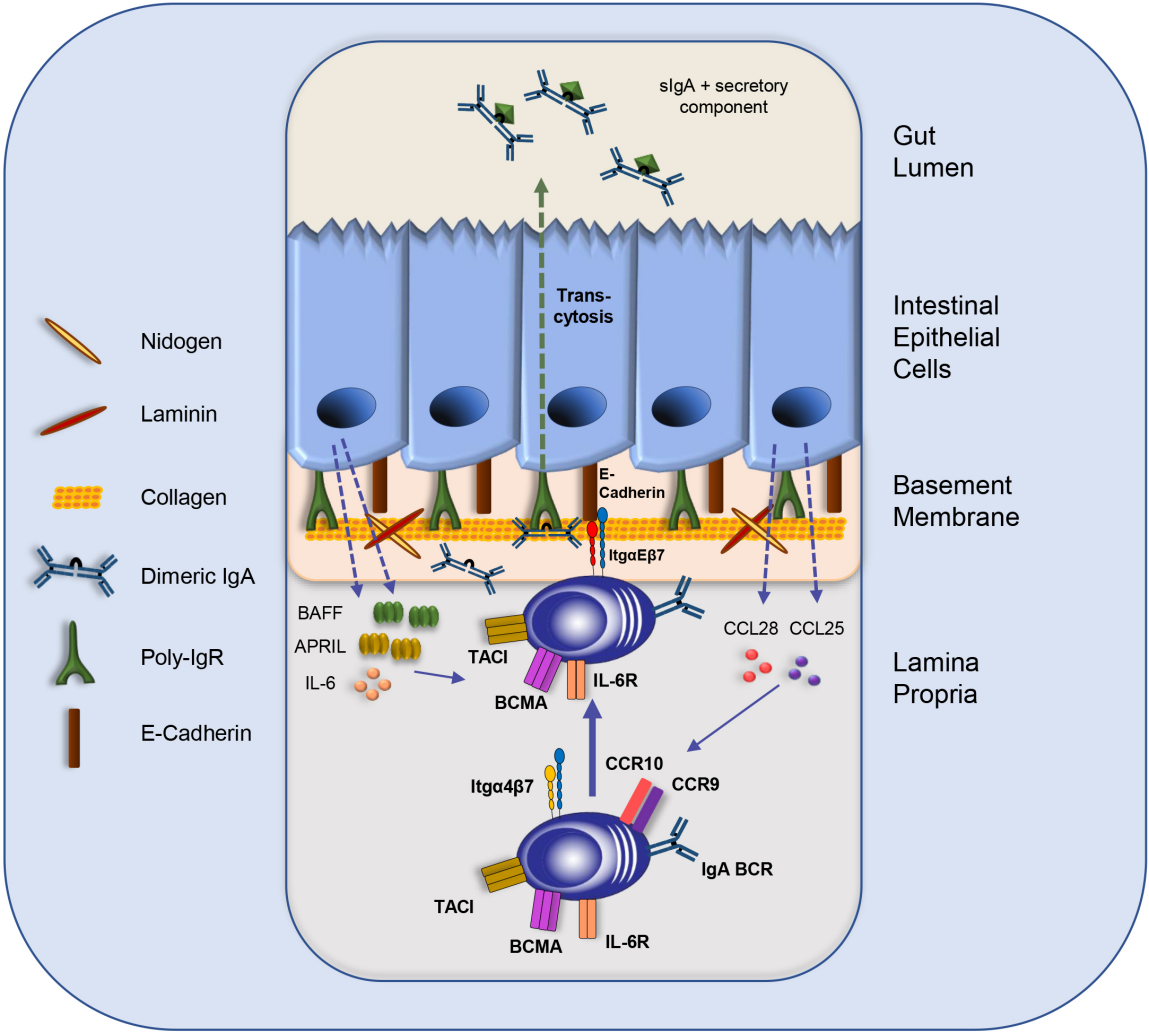
The intestinal IEC-IgA plasma cell niche. IgA plasmablast homing to the LP is directed by integrin α4β7 and the chemokine receptors CCR9 and CCR10. Intestinal epithelial cells (IECs) secrete CCL25 and CCL28 to attract IgA plasma cells and other immune cells. IECs produce the survival cytokines IL-6, BAFF and APRIL. Moreover, the direct interaction of IgA plasma cells with IECs is mediated by the binding of integrin αE to E-Cadherin, which might support plasma cell retention and facilitate the transcytosis of dimeric IgA to the gut lumen *via* binding to the p-IgR on IECs. Furthermore, IgA-secreting plasma cells might interact with components of the basement membrane, such as Collagen type IV and other extra cellular matrix (ECM) compounds. BCR: B cell receptor; IL: Interleukin; Itg: integrin; sIgA: secretory IgA.

Immune cell homing to the intestinal LP is controlled by the timely and spatially coordinated action of specific selectins, chemokine receptors and integrins (59). Gut homing requires the expression and activation of integrin α4β7 which binds to its ligand mucosal addressin cell adhesion molecule-1 (MadCAM-1) on endothelial cells in the high endothelial venules (HEVs) in the PPs and the mLNs, as well as on post-capillary venules in the intestinal LP (60–64). MadCAM-1 is also expressed in lactating mammary glands, the spleen and the bone marrow (65–72). In addition to integrin α4β7 expression, homing to the PPs and the mLNs requires L-Selectin (CD62L) (60, 73). Homing to the gut LP is orchestrated by the chemokines CCL25 and CCL28 and their corresponding chemokine receptors CCR9 and CCR10 on immune cells (**Figure 2**) (35, 74). IECs are the source of the chemokines CCL25 and CCL28 and are therefore crucial for the recruitment of immune cells to the LP, including IgA plasmablasts (35, 74). The critical role of CCR9 was demonstrated in CCR9-deficient mice, in which a severe impact on IgA plasmablast homing to the small intestine was observed (75). In addition to CCR9, CCR10 and its ligand CCL28 contribute to IgA B cell and IgA plasmablast homing. Surprisingly, CCR10-deficient mice had normal serum and fecal IgA levels and exhibited only slight alterations in IgA-secreting cell numbers in the gut LP. However, a striking reduction of IgA-secreting cells was detected in the lactating mammary gland, demonstrating that CCR10 plays a critical role in mammary gland plasmablast homing (76).

We have identified the transcription factor Krüppel-like factor 2 (KLF2) as a key regulator of integrin α4β7 and CCR9 expression and consequently as an essential regulator of IgA plasmablast homing to the intestinal LP. B cell-specific deletion of KLF2 resulted in reduced integrin β7 and CCR9 expression and subsequently in a severe reduction of IgA plasmablasts/plasma cells in the LP concomitant with reduced IgA in the serum and in the gut lumen (77, 78). Besides CCR9 and integrin β7, KLF2 also activates L-Selectin (CD62L) expression in B cells and IgA plasma cells (77–79). Of note, KLF2 directly binds to the promoter of the integrin β7 gene and activates its expression (80). Deletion of integrin β7 in mice resulted in smaller PPs and fewer IELs, reduced numbers of IgA B cells, IgA plasma cells and CD4^+^ T cells in the LP due to impaired homing (81). Moreover, deregulation of the integrin β7 chain was also found in Kmt2d-defienct mice (82), a model for the Kabuki syndrome. The abundance of integrin β7 was reduced on Kmt2d-deficient B cells and consequently, Kmt2d-deficient mice displayed decreased serum IgA levels, smaller PPs and reduced numbers of IgA-secreting cells (82).

## IgA plasma cell interaction with the intestinal epithelium

The intestinal epithelium is the source of the key chemokines CCL25 and CCL28. Both are crucial for attracting immune cells, including B cells and plasmablasts, to the LP (**Figure 2**). Furthermore, direct interactions between epithelial cells and plasma cells were observed. IgA plasmablasts in the LP can be divided into different subsets according to their expression of α4β7 and αE(CD103)β7 integrins. In a recent study, Guzman and colleagues revealed that a subpopulation of IgA plasmablasts in the LP express integrin αEβ7 allowing them to interact physically with IECs *via* E-Cadherin (**Figure 2**). This intimate plasma cell-IEC interaction allows the efficient transcytosis of dimeric IgA *via* the poly-IgR (p-IgR) on IECs to the gut lumen (83). Dimeric IgA consist of two IgA monomers that are covalently connected by the joining chain (J-chain) (84, 85). P-IgR binds dimeric IgA *via* its J-chain and facilitates its transport through epithelial cells by a process which is called “transcytosis” (**Figures 1**, **2**) (22, 23, 84, 85). Upon transport to the luminal side, proteases cleave the ectodomain of the p-IgR. The ectodomain (secretory component) is released together with dimeric, secretory IgA (sIgA). The secretory component protects IgA from degradation and has immunomodulatory functions [**Figure 2** (86)].

Upregulation of integrin αE is a common mechanism applied by immune cells to achieve tissue residency (e.g., DCs, IELs, tissue-resident memory T cells (18–20, 87). Induction of integrin αE expression in IgA plasma cells might be triggered by TGF-ß and RA, both of which are secreted by the IECs.

Therefore, IECs do not only orchestrate the recruitment of IgA plasmablasts to the LP, but also foster the establishment of an intimate IgA plasma cell - IEC interaction. Tissue-residency of immune cells is also regulated by inhibition of sphingosine-1-phosphate (S1P)-mediated migration to blood vessels and lymph. Tissue-resident cells upregula te CD69, which binds to the S1P-receptor 1 (S1PR1) resulting in S1PR1 inhibition and degradation (88); a mechanism that might also apply to IgA plasma cells in their intestinal niche. However, it remains unclear how IgA plasma cells establish the direct interaction with E-Cadherin on IECs in the presence of the basement membrane. Pore sizes in the basement membranes vary and can reach a diameter of up to 8 µm. Pores with sizes of 1-5 µM were frequently found in the follicle-associated epithelium, whereas large pores were mainly found in the crypt region (4). Intra-epithelial lymphocytes can migrate through these pores due to their small cell size (i.e., IELs) or form protrusion (i.e., DCs). If and how plasma cells with their cell sizes ranging from 15-20 µM can migrate through the basement membrane pores remains unclear. One possible scenario is that plasma cells form protrusions through the pores in the basement membrane to establish the binding of integrin αEβ7 to E-Cadherin.

Thus, the β7 integrin chain plays a central role in IgA plasma cells homeostasis in the gut by controlling the migration from IgA plasmablasts from the blood to the LP when bound to the α4 integrin chain, and by enabling the direct interaction of IgA plasma cells with the epithelium when paired with the αE integrin chain (**Figure 2**). How the expression of the αE integrin chain is induced in IgA plasma cells remains unclear. Studies with T cells suggested a role of TGF-β in inducing integrin αE expression (89).

In addition to the supportive function of dimeric IgA transport to the gut lumen, IECs also promote the survival of plasma cells. IECs and smooth muscle cells in the LP were shown to produce IL-6 (90). The role of IL-6 for plasma cell survival is controversially discussed. On the one hand IL-6 supports plasma cell generation (91–94), on the other hand, plasma cell short-term survival after transfer into IL-6-deficient mice is not impaired (95). For their generation and survival, plasma cells require the expression of cytokine receptors TACI and B cell maturation antigen (BCMA) on their cell surface (57, 96–100). BCMA might exert its pro-survival effect by upregulating the anti-apoptotic factor Mcl-1 as shown for bone marrow plasma cells (101). IECs are one source of the ligands for these survival receptors, i.e., APRIL and BAFF (56, 58). Thus, regarding their biological properties, IECs are the central component of the intestinal plasma cell niche, comparable to the stromal cells in the plasma cell survival niche in the bone marrow (55, 64). Furthermore, IEC-derived TSLP fostered production and secretion of APRIL and BAFF by myeloid cells, such as DCs (29, 58). APRIL production by intestinal eosinophils was also reported and might contribute to IgA plasma cell survival (102). Thus, IECs together with various immune cells, such as myeloid cells, T cells and eosinophils provide cytokines as well as cell-cell contact that allow IgA plasma cells to persist for many decades in the human intestine and to become long-lived in the murine intestine (103–105). Therefore, LP cells in cooperation with epithelial cells may constitute a plasma cell survival niche similar to the one described for the bone marrow. In both microenvironments, plasma cells are provided with the survival factors APRIL and IL-6 by neighboring cells. Furthermore, the integrin-mediated cell-cell contact of plasma cells with bone marrow stromal cells is a major aspect contributing to the survival of long-lived bone marrow plasma cells (55, 64, 106, 107). In the intestinal niche, IgA plasma cells also express integrins that mediate their homing to the LP as well as their adhesion to e.g., IECs *via* integrin αEβ7; an interaction that might support the persistence of IgA plasma cells and the long-lasting secretion of protective IgA. However, due to the high turn-over rate of IECs, the constant process of epithelial cell death, and their dynamic replacement, it remains unclear how IgA plasma cells in the proximity or in direct contact with the epithelium achieve longevity. Furthermore, it remains elusive how the plasma cell survival niche in this highly dynamic environment is preserved. Hence, kinetic studies combined with spatial analyses of IgA plasma cells in their intestinal survival niches and in different sections of the intestine (i.e., small intestine, colon, and cecum) and within different areas along the crypt-villus axis need to be performed.

## The impact of nutrients on the intestinal epithelium and IgA plasma cells

### The Aryl hydrocarbon receptor: Structure and ligands

The intestinal immune system can be influenced by various external factors, e.g., the diet (108). Therefore, it is more than likely that the modeling of the intestinal plasma cell survival niche and the support of plasma cell function does not depend only on the intestinal milieu itself. One transcription factor, the Aryl hydrocarbon receptor (AhR), has been described to connect nutritional intake with the microbiota composition and the immune response (109). The AhR was first discovered as a sensor for xenobiotics like polycyclic aromatic hydrocarbons (PAHs) and dioxins (110). The treatment of Hepa-1 cells with 2,3,7,8-tetrachlorodibenzo-p-dioxin (TCDD) induces the translocation of the AhR from the cytosol to the nucleus, followed by the induction of drug-metabolizing enzymes (111). Therefore, AhR was revealed to function as a transcription factor involved in metabolic processes and detoxification early after its discovery. Today, we know that the AhR is a member of a transcription factor superfamily mainly characterized by two motifs, a basic N-terminal helix-loop-helix (bHLH) and a Per-Arnt-Sim (PAS) domain with two subunits (PAS-A and PAS-B) (112). While the bHLH domain allows dimerization of the protein and its binding to DNA, the PAS domain located at the C-terminal end of the bHLH domain is necessary to bind other PAS-proteins like the aryl hydrocarbon receptor nuclear translocator (ARNT) and the chaperone family member heat-shock protein 90 (HSP90) (113, 114). Essential and unique for AhR among the bHLH/PAS superfamily members, its PAS-B subdomain encodes for a ligand binding side specific for “all classes of dioxin receptor ligands”, which partially overlaps with the HSP90 binding side (115). The nuclear localization and the nuclear export signal can be found at the N-terminal side of the AhR protein, respectively (116). In contrast, the C-terminal region contains a glutamine-rich transactivation domain (TAD), which is essential for binding the AhR to its transcriptional co-activators and, therefore, for the induction of its target genes transcription (117). In the absence of AhR ligands, HSP90 binds to the PAS domain, thereby restricting its entrance to the nucleus and retaining AhR in an inactive conformation in the cytosol. Ligand binding to the PAS-B domain of the AhR in the cytosol results in the dissociation of HSP90, where after the AhR ligand complex translocates into the nucleus, binds to various transcriptional co-activators and induces its target gene expression (118). Important AhR-targets are the enzyme Cyp1a1 that degrades xenobiotics serving as AhR ligands (119), and the AhR repressor (AhRR) that suppresses AhR-activity by competing for the binding to the AhR co-activator ARNT (120). Both mechanisms result in a feedback loop regulating AhR activity. AhR-ARNT complex binding to the xenobiotic response element (XRE; also dioxin response element, DRE) in the target gene promotor region and its mediated gene expression is defined as the canonical AhR signaling (121–123). However, more recent studies demonstrated that AhR can interact with a variety of co-activators in a non-canonical signaling pathway, depending on the co-activator’s availability, and presumably the nature of the activating ligand (124, 125). Therefore, all studies addressing the AhR function by activation or inhibition through specific ligands must be compared carefully to allow reliable conclusions.

Among the initially described toxic chemicals functioning as AhR ligands, a vast number of naturally occurring (126) and synthesized AhR agonists and antagonists have been described (125, 127). The number of the latter is steadily increasing with the rising interest in AhR as a drug target in various diseases, including cancer, rheumatoid arthritis and inflammatory bowel disease (125). The interplay between the dietary composition, the variety of diet-derived AhR ligands generated by individual members of the intestinal microbiota or liver enzymes and the transport of these compounds through the organism to the site of action is incredibly complex and differs between individuals. Therefore, the prospect of treating diseases by directly manipulating AhR-activity through specifically designed ligands administrable as drugs seems promising. However, the possibility of treating or preventing diseases or even supporting our immune defenses with a diet rich or low in AhR ligands is tempting, especially as a mixture of AhR ligands can result in an altered AhR-functionality compared to their individual activity (128).

A wide range of naturally occurring, exogenous AhR ligands are generated during the metabolism of tryptophan or glucobrassicin found in vegetables (129–131). Metabolism of tryptophan by individual microorganisms of the intestinal microbiota results in the production of the AhR agonists indole acetic acid (IAA), indole-3-acetaldehyde and indole-3-aldehyde (IAld) (132–134). In addition, a very potent AhR agonist is the tryptophan photoproduct 6-formylindolo [3,2-b] carbazole (FICZ), which is generated by UV-radiation (135). In contrast to tryptophan, glucobrassicin from cruciferous vegetables of the family *Brassicaceae* is already metabolized in the oral cavity by myrosinases, resulting in the production of the AhR agonist precursor indole-3-carbinol (I3C) and indole-3-acetonitrile (I3ACN) (136). In the stomach, non-enzymatic acid condensation of I3C and I3ACN produces chemical compounds, such as 3,3’ diindolylmethane (DIM), 2-(indol-3-ylmethyl)-3,3’ diindolylmethane and indolo[3,2-b]carbazole (ICZ), that can function as AhR ligands (131, 137–139).

Diet-derived short-chain fatty acids (SCFAs) like butyrate can also induce AhR and AhR target gene expression in human IECs (140). This is particularly interesting as SCFAs are discussed for preventing or curing different diseases (141). In addition, by consuming stimulants like coffee (caffeine) or tobacco (nicotine), the AhR can be activated in the corresponding organs, while flavonoids found in, e.g., tea function mainly as AhR antagonists (142–144).

Even though studies of human and murine blood plasma showed biologically active concentrations of diet-derived AhR ligands that can potentially control AhR activity in distinct organs (145–148), the digestive tract is the first responding organ that is in contact with the highest concentrations of AhR ligands due to the primarily oral uptake of AhR ligands or their precursors.

### The Aryl hydrocarbon receptor and its function in the intestinal tract

AhR protein abundance has been identified in various cell types, including a wide range of immune cells as well as cells functioning at barrier sites like the lung, skin or intestine (149). Upholding intestinal homeostasis requires a delicate interplay between the microbiota commensals, the IECs, and immune cells found in the LP or IELs, with the majority expressing AhR in varying abundance (150). The analysis of an AhR-reporter mouse model revealed widespread expression of AhR in the intestinal epithelium with a proximal-distal gradient in the small intestine (151). A weaker and mosaic-structured AhR expression was detected in the colon of the same mice, indicating a more cell-type selective AhR function. Of note, the AhR gradient in the intestinal tract is comparable to the observed IgA gradient (1, 2). Therefore, AhR signaling may play a critical role in the intestinal plasma cell niche.

Of note, depending on the sensitivity of the used method, a gene expression analysis in whole organs does not always allow reliable distinguishing between different cell types. For this reason, more specific and detailed studies are required to identify the cell types in the intestinal tract that express AhR and to reveal how these cells are influenced by AhR activity. Therefore, advanced technologies like spatial transcriptomics will help to solve the remaining questions about the intestinal plasma cell niche and the functional relevance of AhR signaling therein.

However, several studies addressed the role of AhR, or more precisely of individual AhR ligands, for the intestinal epithelial barrier. One study revealed that FICZ supplementation prevents the intestinal permeability caused by damage of the epithelial layer due to decreased tight junction stability between epithelial cells during dextran sodium sulfate (DSS)–induced murine colitis (152). The authors demonstrated that FICZ-induced AhR signaling in a Caco-2 cell monolayer suppressed NF-kB p65 signaling and thereby protected against the tumor necrosis factor (TNF)-α/IFN-γ-mediated reduction in tight junction protein. However, FICZ-mediated rescue of DSS-colitis *in vivo* may not exclusively be attributed to the intrinsic role of AhR in IECs, as the activation of AhR also affects immune cells in the intestinal tissue (153). Supporting the importance of AhR in IECs, AhR-deficient mice or mice lacking AhR specifically in IECs were more susceptible to infection with the gram-negative bacterium *Citrobacter rodentium* (154–156). This phenotype could be re-produced by constitutive expression of the AhR-target Cyp1a1 (R26^Cyp1a1^-mice) (109). In this mouse model, constant degradation of diet-derived AhR ligands by Cyp1a1-overexpression efficiently inhibited AhR signaling in IECs, resulting in an impaired immunity to *C. rodentium* and, thereby, an increasing systemic burden. However, the dietary supply of the AhR agonist precursor I3C enabled sufficient clearance of *C. rodentium* and rescued R26^Cyp1a1^-mice.

Bacterial invasion of the LP does depend on the permeability of the IECs. Still, it is also controlled by the thickness of the intestinal mucus layer and its abundance of AMPs (**Figure 1**). Therefore, an increased bacterial burden in AhR-deficient mice during a *C. rodentium* infection may also be caused by a diminished intestinal mucus layer. This hypothesis is supported by the observation that AhR is required for the differentiation of secretory cells in the intestinal epithelium, thereby enhancing the resistance to enteropathogenic *Escherichia coli* (157). In detail, activation of AhR by L-Kynurenine represses Notch1 signaling in murine intestinal cells, thereby allowing indoleamine 2,3-dioxygenase 1 (IDO1)-mediated promotion of differentiation into goblet and paneth cells. Furthermore, IEC-specific AhR-deficiency restricted stem cell differentiation to epithelial cells, e.g., goblet cells, while stem cells proliferated uncontrollably, resulting in an increased tendency to malignant transformation (154).

Studies in human colorectal cancer showed that upregulation of Wnt signaling at the bottom of the intestinal crypts through mutations in the Wnt-pathway component genes adenomatous polyposis coli (APC), β-catenin and/or AXIN2 causes this pathogenicity (158). Therefore, it is of particular interest that, besides its functions as a transcription factor, AhR was shown to support ubiquitination and, thereby, degradation of β-catenin by participating in an E3 ubiquitin ligase complex (159, 160). In addition, analyzing mice lacking AhR specifically in IECs demonstrated that dietary-induced AhR signaling prevents intestinal tumorigenesis by inhibiting the Wnt/β-catenin signaling in intestinal stem cells through induction of the transcription of E3 ubiquitin ligases (154). Furthermore, AhR was shown to be indispensable for the termination of the regenerative response in IECs after an injury (161). Here, the authors analyzed colonic organoid cultures under specific media conditions that either simulate differentiating or regenerative conditions. They demonstrated by RNAseq analysis that AhR regulates critical factors involved in the regenerative response or the reacquisition of intestinal identity post-injury. Interestingly, AhR signaling was also shown to control the differentiation of hematopoietic stem cells into lymphocytes (162). In addition, AhR can directly function as a tumor suppressor of acute myeloid leukemia by suppression of self-renewing of leukemia stem cells (163).

PCB 126, formerly produced as a lubricant in electronic equipment (164), is a very toxic AhR ligand (165). Treatment of human breast epithelial cell cultures with a very low dose of PCB 126 resulted in a significant reduction of CCL28 mRNA levels (166). As the parallel treatment with an AhR inhibitor nullified the observed phenotype, the authors concluded that AhR activation by PCB 126 inhibits CCL28 expression in an AhR-dependent manner. CCL28 secretion by IECs is associated with the homing of IgA plasmablasts to the small intestine (64, 74) (**Figure 2**). Therefore, it should be considered as an AhR-controlled mechanism associated with antibody-secreting cell (ASC) migration in the gut. Of note, PCB126 treatment also altered the microbial community structure in the murine intestines (167).

Additional studies in AhR- or IL-10-deficient mice demonstrated that treatment with the *Braccicacea*-originating AhR ligand indole-3-carboxaldehyde (ICA) promotes goblet cell differentiation and proliferation of IECs through IL-10 in an AhR-dependent manner (168). It is well known that aging results in changes in the intestinal epithelium composition that involves the reduction of goblet cells, thereby, a decline in the epithelial barrier integrity and increased risk of inflammation. However, early colonization with bacteria known for their capacity to produce indoles, dampens this age-related phenotype. Furthermore, another xenobiotic AhR ligand called 2,3,7,8-tetrachlorodibenzofuran (TCDF) shifted the ratio of *Firmicutes* to *Bacteroidetes*, a change in the microbial community which was associated with the development of intestinal diseases (169). As the described phenotypes did not occur in AhR-deficient mice, the authors concluded that they are AhR-dependent.

In another study, the analysis of liver cells from TCDD-treated mice showed that AhR can potentially induce the expression of E-Cadherin when interacting with KLF6 as a co-activator (170). As E-cadherin may be necessary for cell-cell interaction between IECs and lymphocytes in the intestine (83), this mechanism has to be considered when analyzing the role of AhR in the intestinal plasma cell niche. However, functional studies are still missing, and it must be determined whether AhR interacts with KLF6 to induce E-Cadherin in IECs.

Interestingly, mice suffering from 2,4,6-trinitrobenzenesulfonic acid (TNBS)-induced colitis showed significantly less damage in colonic tissue and reduced infiltration of inflammatory cells when treated with the AhR ligand TCDD prior to disease onset (171). Surprisingly, the treatment with TCDD led to an increased mRNA abundance of the plasma cell survival factor APRIL in colonic tissue three days after TNBS-induction. As the colon harbors a vast variance of cell types, the localization of the exact APRIL source seems challenging and could be addressed by spatial transcriptomics. However, *in vitro* LPS-activated IECs showed a profound increase in APRIL and BAFF mRNA when treated with TCDD. Therefore, TCDD-induced AhR signaling in IECs may support the viability of ASCs in the intestinal survival niche by inducing the secretion of the survival factors APRIL and BAFF (**Figure 2**).

Based on these data, it is out of the question that AhR signaling plays a vital role in IECs. However, if AhR-regulated processes in IECs are essential for the intestinal plasma cell niche and healthy IgA secretion remains unanswered.

### The function of the Aryl hydrocarbon receptor in immune cells of the intestinal niche

#### Dendritic cells

Mice with a CD11c-Cre-mediated specific deletion of AhR in DCs suffer as much from DSS-induced colitis as AhR^-/-^ mice (172). Surprisingly, the development of the intestinal epithelium was disrupted in these mice, resulting in shorter villi, reduction in paneth cells, but an increase of goblet cell numbers per villus. Therefore, AhR signaling in DCs may be essential for the intestinal plasma cell niche. Interestingly, AhR-deficient CD11c^+^MHCII^high^ DCs in the mLNs expressed less integrin αE (CD103), potentially affecting their migration behavior in the intestinal tissue and indicating that CD103 is a potential AhR target gene. As CD103 might be involved in IgA plasma cell homing to the intestinal tissue and their interaction with IECs, this mechanism is of potential interest when determining their intestinal survival niche. Of note, IgA levels did not differ between the feces of mice lacking AhR in DCs and of control animals.

Culturing human monocytes in a specific differentiation culture system was shown to induce differentiation into monocyte-derived (mo-) macrophages and mo-DCs which resemble those found *in vivo* (173). Interestingly, under these culture conditions FICZ-induced AhR signaling favored the differentiation into mo-DCs by directly inducing the expression of the transcription factor Blimp-1. As Blimp-1 is the key transcription factor driving B cell differentiation into ASCs, an AhR-Blimp-1 axis would be particularly interesting for the formation of IgA plasma cells.

### Macrophages

AhR-deficient LPS-activated macrophages secreted elevated amounts of IL-6, IL-12, and TNF-α (174). In detail, AhR mediates inhibition of IL-6 promotor activity by forming complexes with NFκB and signal transducer and activator of transcription 1 (Stat1). Furthermore, LPS-induced inflammation of bone marrow stromal cells *in vitro* results in an increase of IL-6 (175). However, this was abolished when cells were pre-treated with the AhR ligand TCDD. As IL-6 supports human long-lived plasma cells, AhR-controlled IL-6 production in the intestinal environment could play an important role in the intestinal plasma cell survival niche (93).

### ILCs

Nutrition studies demonstrated that a dietary lack of the AhR ligand precursor tryptophan alters the gut microbiota and results in an impaired intestinal immune system (176). Especially tryptophan degradation to indole-3-aldehyde by *Lactobacilli* induces AhR signaling in ILC3s, thereby promoting the production of IL-22 (132), an essential mechanism described in the immune response to *Citrobacter rodentium* (177). Furthermore, IL-22 is indispensable for a functional intestinal epithelial barrier, as it mediates the differentiation of intestinal stem cells into IECs and supports their viability (178). Thereby, IL-22 guarantees mucosal protection from damage and the repair of defects in the epithelial barrier integrity. Interestingly, IL-22 is expressed in the small intestine but barely in the colon (179), forming a gradient along the intestinal tract comparable to the one for AhR expression and IgA density. Thus, AhR-mediated IL-22 production may be beneficial for IgA plasma cell longevity in the intestine.

In contrast, the numbers of IL-5 and IL-13-secreting ILC2 cells were significantly elevated in AhR-deficient mice (180). Authors claimed that the AhR is the regulating factor that balances the intestinal ILC2 and ILC3 populations and, thereby, the intestinal immune homeostasis with an appropriate response to acute infections. As the function of ILCs in the intestinal tract is out of the question, it seems obvious to assume that they are also an AhR-regulated part of the intestinal plasma cell niche.

### T cells

A variety of T cell subpopulations express AhR (181). However, there are contradicting reports about the abundance and importance of AhR in the same T cell subpopulations. AhR was shown to induce IL-22 production in CD4^+^ T cells by interacting with Stat3 as a co-activator (182). IL-22 is secreted by Th17 and Th22 cells, both of which require the activity of the transcription factor RORγt for differentiation (183–185). Of note, more recent studies revealed that AhR and RORγt can directly interact as a transcription factor complex (186) and are a potential target for treating lupus erythematosus (187). However, *in vitro*-activated T cells, primed for differentiation into Th17 cells, showed even further elevation of IL-22 production when treated with the AhR agonists β-naphthoflavone and FICZ (188). In strict contrast, AhR^-/-^ mice showed increased numbers of Th17 cells and elevated secretion of the cytokines IL-22 and IL-17 compared to AhR^+/-^ mice after imiquimod-induced psoriasiform skin inflammation (189). With IL-22 being a critical cytokine in the intestinal environment, the AhR-T cell interaction must be kept in mind when analyzing the role of nutritional AhR ligands and their impact on the intestinal IgA plasma cells niche.

Analysis of a Foxp3^Yfp-Cre^ reporter mouse demonstrated that Tregs located in the spleen and mLNs showed less AhR expression than intestinal Treg cells (190). The authors of this manuscript also pointed out that AhR has a cell-intrinsic role in intestinal Tregs. They demonstrated that AhR-deficient intestinal CD4^+^TCRβ^+^Foxp3^+^ Tregs lack the expression of CD103 and the chemokine CCL20, which is involved in cell migration. CD103 expression in Tregs is important for their activation and retention at the site of inflammation and CD103 can also be found on IgA plasma cells (83). Furthermore, mice treated with the AhR agonist FICZ expressed more CD103 in intestinal Tregs than control-treated animals, implying CD103 as a potential AhR target gene. As AhR-deficient intestinal Tregs express more inflammatory cytokines like IL-17 and IFN-γ, this study demonstrated the importance of AhR signaling in Treg cells for intestinal homing, and its requirement for their immunosuppressive function in colitis. T cells as a part of the intestinal micro-milieu regulate inflammatory responses and also the differentiation of epithelial cells. Therefore, T cells may undoubtedly be an AhR-controlled part of the intestinal plasma cell niche.

## The role of AhR in B cells, plasma cells, and IgA production

### B cells

Various studies addressed the effect of AhR ligand treatment on B cells (153). Few of them focused on the intrinsic function of AhR in B cell activation and differentiation, and none discussed the role of AhR in intestinal plasma cells. However, the results of these studies are controversial, claiming either a supportive function of AhR during B cell activation or the exact opposite, i.e., inhibition of the B cell response. A study comparing activated human and murine B cell cultures claimed that TCDD treatment diminished IgM secretion *in vitro*, independent of the species (191). However, intracellular IgM abundance was reduced in activated murine B cells, but increased in human B cells after TCDD treatment. This indicates that TCDD: AhR inhibits the formation of IgM-secreting cells, even though the data are partially contradicting. There may also be ligand- or species-depending differences in AhR function in B cells.

Investigations of the role of AhR signaling in the B cell response *in vivo* using an adoptive transfer model demonstrated that AhR^-/-^ mature B cells are outcompeted by AhR^+/+^ cells in the bone marrow and mLNs of mixed bone marrow chimeras (192). Even more interesting, challenging a recipient mouse reconstituted with AhR^-/-^ and AhR^+/+^ splenocytes revealed that AhR-deficient B cells expanded less. Of note, B cell-specific AhR-deficient mice showed altered steady state serum Ig titers compared to controls, with elevated IgM but diminished IgG1 concentrations. IgA serum titers were unaltered. ASC numbers were reduced in the spleen of the same mice, but remained unchanged in their bone marrow. These data indicate a function of AhR in ASC formation, but very likely not in their maintenance in the bone marrow. Unfortunately, this study failed to determine ASC formation and maintenance in other organs or niches, e.g., the intestinal tract or their migration behavior. Even though IgA serum titers were unvaried in these mice, changes in intestinal plasma cells or the amount of mucus secreted IgA was not addressed and remains unclarified. However, this study indicates that AhR signaling may also be necessary for the formation of intestinal plasma cells.

One study analyzing B cell-specific AhR-deficient mice demonstrated that AhR signaling in regulatory B cells (Bregs) induces IL-10 secretion, thereby, dampening inflammatory Th1 and Th17 responses in a murine rheumatoid arthritis model (193). A more recent study of the same group indicates that AhR signaling induced by dietary SCFA butyrate supplementation favors the formation of Bregs during GC response and, thereby, alleviating inflammation in the cause of rheumatoid arthritis (194). In sharp contrast to the previously mentioned study (192), the authors claimed that butyrate-induced AhR signaling in B cells suppresses GC formation and plasmablast differentiation. But even though butyrate treatment reduced the numbers of CD19^+^ plasmablasts in the spleen of control animals, there was a significant increase in this population detected in butyrate-fed B cell-specific AhR-deficient mice. In contrast, numbers were unaltered between standard-fed knock-out and control animals. As B cells lack AhR in this model, why did butyrate still affect plasmablast numbers in the knock-out mouse? In addition, the population defined as Bregs showed a severe upregulation of the plasma cell signature transcription factor Blimp-1 (*prdm-1* gene) when lacking AhR and butyrate feeding markedly reduced Blimp-1 expression independent of the genotype. This gene expression profile questions the nature of the analyzed cell population, especially as plasma cells can also secrete IL-10 in autoimmune inflammation (195) and express high amounts of CD24 (196, 197). Of note, the analysis of the B cell response in the intestinal tract was missing in this study.

Due to contradicting data, the role of AhR signaling in B cell activation and plasma cell differentiation is still unresolved. Especially the function of AhR in the intestinal ASC pool, and their migration behavior remains completely unclear.

### IgA production and secretion

In addition to several studies that described a potential function of AhR signaling in B cells, there are also analyses addressing a connection between AhR activity and IgA production.

Analyzing the effect of TCDD treatment during viral airway infection with Influenza A demonstrated an overall immunosuppressive phenotype (198). Interestingly, virus-specific IgG and IgM levels were reduced in the blood plasma of TCDD-treated infected mice, while antigen-specific IgA was 4-fold increased. In contrast, analyzing broncho-alveolar lavage fluid revealed that IgA was unaltered at the side of infection, even so, IgG levels were again diminished after TCDD application. Of note, all mice in this study were female and observations may reflect a gender-specific phenotype. Even though this data may indicate a connection between AhR signaling and IgA production, the authors failed to determine whether this phenotype was associated with a B cell-intrinsic AhR signaling or a secondary effect due to the overall observed alterations in T cells and cytokine production. Furthermore, organ-specific alterations in IgA levels indicate a differential role of AhR activity for secreted, mucus-associated IgA and systemic IgA in the blood. This contradicts a potential function of AhR signaling in p-IgR-mediated IgA transport through lung epithelial cells into the mucus. However, whether the same is true for the intestinal tract remains unanswered.

Research focusing on the formation of IgA-secreting cells in the intestine revealed that feeding wildtype C57Bl/6 mice with the AhR ligand TCDD alters fecal IgA concentrations independent of the circadian rhythm (199). However, the authors did not clarify whether the observed phenotype is mediated by a B cell-intrinsic AhR function or associated with AhR activity in the surrounding cells e.g., IECs. Furthermore, TCDD treatment elevated fecal and serum IgA levels in male mice independent of the analyzed dose. However, female mice showed reduced IgA levels when treated with low doses of TCDD, while results were comparable to their male counterparts during high-dose TCDD treatment. Notably, hormonal changes can affect IgA secretion (200). Regarding the female menstrual cycle, the AhR can recruit the estrogen receptor α to the promotor region of AhR-regulated genes (201) or even to the estrogen response elements (202). Therefore, gender-specific variances in AhR ligand-induced IgA secretion are not surprising and must be addressed in future experiments.

In support, AhR ligand 2,3,7,8-tetrachlorodibenzofuran (TCDF)-fed male wildtype mice showed increased fecal IgA abundance and elevated concentrations of inflammatory cytokines in the ileum (169). Surprisingly, LPS abundance in the blood serum was significantly increased in TCDF-treated mice, indicating diminished intestinal barrier integrity and increased translocation of bacteria from the gut lumen into the LP and adjacent organs. These data oppose previously described studies which showed that FICZ-mediated AhR activity in IECs strengthened intestinal barrier function by increasing tight junction protein production (152). This conflicting data may be caused by the authors missing to address the question if the TCDF-mediated disruption of the intestinal barrier is AhR-dependent or caused by the very toxic nature of the ligand. In addition, it has to be considered that altered IgA levels in TCDF-fed mice could be triggered by increasing bacterial infiltration and, therefore, may not depend on TCDF-induced AhR activity in ASCs.

A study assessing TCDD-mediated AhR signaling and its influence on the course of Crohn`s disease demonstrated faster recovery of TCDD-fed mice from 2,4,6-trinitrobenzenesulfonic acid (TNBS)-induced colitis, which was associated with increased numbers of Foxp3^+^ Tregs in the intestines (171). Interestingly, IgA concentrations significantly increased in feces and in homogenized colon tissue after TCDD treatment. This phenotype was independent of TNBS-induced colitis but mediated by AhR activity, as IgA levels were comparable between TCDD- and control-fed AhR^-/-^ mice. As mentioned before, TCDD treatment of LPS-activated IECs led to elevated APRIL and BAFF mRNA abundance, cytokines known to support B cell and ASC viability, respectively. In addition, TCDD-induced AhR signaling in IECs or intestinal immune cells could trigger the production of cytokines that induce or support ASC differentiation. Therefore, augmentation of IgA levels in TCDD-treated mice may rather be attributed to AhR signaling in other cell types and not B cell or ASC intrinsic.

A unique study among AhR-focused research projects determined the influence of AhR activity on IgA production in neonatal mice (203). Therefore, researchers compared AhR^-/-^ mice and wildtype mice whose parents were already fed with a standard diet or an AhR ligand-free diet, which was continued after birth. Two-week-old AhR^-/-^ mice showed no fecal IgA, while their serum IgA levels were comparable to wildtype mice fed with a standard diet. In contrast, an AhR ligand-free diet abolished serum IgA in wildtype mice but did not affect fecal IgA concentrations. Furthermore, the authors claimed that IgA abundance in the gastric content were comparable between animals during the nursing period, indicating no influence of maternal IgA on the observed phenotype. However, a direct diet-mediated effect on 2 week-old suckling mice is hard to imagine. Therefore, it has to be considered that other components in the maternal milk besides IgA itself may contribute to the observed differences in the IgA concentrations in the different groups of neonatal mice. Interestingly, depending on the available diet for nutrient intake, eight-week-old weaned mice did not show any differences in serum IgA concentrations. In contrast, only mice fed with an AhR ligand-free diet lacked fecal IgA, likely caused by reduced CD19^+^B220^+^ B cell numbers in the GALT. Even though the authors provided a unique set of data analyzing the influence of a diet or AhR signaling in neonatal mice, the results of this study are contradicting. As the observed alterations in IgA concentrations differed between AhR ligand-free diet-fed mice and AhR^-/-^ mice, these results implicate an AhR-independent effect of the diet on the intestinal immune system. As obviously all cells of an organism are affected by the deficiency in AhR^-/-^ mice or mice fed with a specific diet, their analysis does not allow conclusions about the B cell-intrinsic role of AhR in the formation of IgA-secreting cells or IgA-secretion itself. However, the differences between fecal and serum IgA concentrations in mice of the same genotype or diet-fed group imply a differential AhR function for secreted mucus-associated IgA and serum IgA, again.

Regarding the significant number of studies addressing the AhR function, it was often neglected that TCDD is a toxic substance causing oxidative stress, DNA damage and endocrine disruption amongst other symptoms (204–207). Until now, it is just partially known which of these mechanisms are caused by TCDD : AhR or an AhR-independent pathway. Therefore, studies analyzing the effect of AhR by simply adding a chemical like, e.g., TCDD to the system must be viewed with caution.

## Spatial transcriptomics as a future approach to address open questions in intestinal IgA plasma cell biology

Novel approaches utilizing spatial transcriptomics (ST) will help to understand how plasma cells differ in their phenotypes and functions in different anatomical locations. ST allows the comparative analysis of gene-expression profiles of IgA plasmablasts and plasma cells in their anatomical and physiological environment. In this context, comparing the transcriptomes of IgA plasma cells in the villus tips to those located next to the crypts in human and murine small intestines will be of interest. ST of intestinal stromal cells along the small intestine villi - crypt axis have already been successfully conducted and resulted in the identification of four spatially distinct mesenchymal cell populations (208). The characterization of the gene expression profiles of IgA plasma cells of the small intestine, the colon, and the cecum under normal and disease conditions, after oral immunizations or upon exposure to specialized diets will provide important insights into the plasticity and heterogeneity of IgA plasma cells and their response to environmental factors. Furthermore, the combination of genetic models (e.g., conditional knockout mice) with ST will allow us to identify the impact of gene function on IgA plasma cell heterogeneity. Hence, ST combined with functional analyses will help to answer the following open questions in IgA plasma cell biology:

1) How can IgA plasma cells persist for decades in the LP in close proximity to the epithelium while IECs are constantly replaced and renewed? The intestinal epithelium is characterized by a high turn-over rate of 3-5 days. Stem cells in the crypts continually divide and give rise to transit-amplifying cells which then differentiate into specialized IECs. During this process, the newly formed cells move from the crypt towards the villus tip. As soon as they reach the villus tip, they undergo cell death and are shed off. The processes of proliferation, differentiation and cell death are regulated by gradients of ligands of the Wnt, the BMP, the Notch, and the Ephrin signaling pathways (7, 8), some of them controlled by AhR signaling. As dividing and differentiating IECs are moving towards the villus tip, it remains elusive how the interaction between IgA plasma cells and IECs is maintained. Moreover, the impact of the Wnt, Notch, BMP, Ephrin or AhR ligand gradients on IgA plasma cell survival and function is unknown. Transcriptional regulation of components of the above-mentioned signaling pathways by e.g., AhR signaling, can be identified by applying ST to IgA plasma cells dissected from different regions within the crypt-villus axis.

2) How does the interaction with the basement membrane and the ECM affect IgA plasma cell survival and functions? IECs reside on a basement membrane consisting of a dense network of ECM components. Therefore, the interaction of IgA plasma cells with ECM components might be crucial for retaining IgA plasma cells in close proximity to the epithelial layer. The basement membrane has been shown to contribute to cell migration and differentiation. In addition, the basement membrane exerts barrier functions (209). It contains pores that allow immune cells (e.g., IELs or protrusions of DCs) to interact directly with epithelial cells and to reside within the epithelial layer. As shown for the rat intestine, pore sizes in the basement membrane are variable. Their numbers differ in areas of the villous structures and in proximity to lymphocyte follicles and M cells (4). How plasma cell homeostasis and plasma cell contact with the components of the basement membrane, the ECM, and the epithelium impacts their survival, their regulatory functions, and IgA secretion is poorly understood and needs to be investigated.

3) How does compartmentalization affect IgA plasma cell and epithelial cell function? The small intestine consists of the duodenum, jejunum, and ileum. The duodenum is characterized by a high concentration of food ligands and a low bacterial content. Along the duodenum–ileum axis, the concentrations of food ligands decrease, the bacterial concentration, however, increases (1, 2). Concomitantly, CCL25 production is higher in the duodenum compared to the ileum, enabling the recruitment of more immune cells. Furthermore, higher numbers of IgA cells are located in the duodenum. Moreover, the expression of the p-IgR is higher in the duodenum resulting in higher luminal sIgA. However, the numbers of IELs decrease from the duodenum to the ileum (1, 2, 210). In addition, expression of AhR also shows a proximal-distal gradient in the small intestine, indicating changes in the impact of food compounds on the transcriptional program of epithelial and immune cells. Hence, IgA plasma cells in the proximal and the distal parts of the small intestine are confronted with varying environmental signals. To determine the impact of these changing conditions, comparative spatial analyses of IgA plasma cells in duodenum, jejunum and ileum are required. Moreover, IgA plasma cells can reside in different locations along the crypt-villus axis within the villi. Depending on their location, they are exposed to gradients of Wnt, BMP, Notch, and Ephrin factors and to a constant change of their neighboring IECs as these cells move towards the villus tip. ST of IgA plasma cells from the crypt, the mid-villus and villus tip area will identify how the anatomical location modulates IgA plasma cell survival and function (i.e., cytokine release). In addition, mucosal surfaces in the small intestine and the colon are structurally very different. In the colon, villi are absent and the epithelial surface is flattened. However, the mucus layer is thicker due to a higher frequency of goblet cells within the colonic epithelium (14). Paneth cells can only be found in the small intestine but are absent in the colon. The bacterial density peaks in the colon concomitant with higher numbers of IgA-secreting cells and higher p-IgR expression compared to the small intestine (1, 2, 210). In humans, IgA-secreting cells can be subdivided in IgA1- and IgA2-secreting cells. IgA1 and IgA2 antibodies differ in their hinge regions (1, 211–213). The shorter hinge region of IgA2 might be more resistant to degradation by bacterial enzymes, and explain the predominant occurrence of IgA2 in the colon and the enrichment of IgA1 in the small intestine. Characterizing the gene expression profiles of human IgA1- and IgA2-secreting plasma cells will identify differences in their homing, survival, and functional abilities. Therefore, comparing IgA plasma cell subsets in different regions of the small intestine to those residing in the colon will provide a deeper insight into the heterogeneity of the IgA plasma cell pool.

4) How do signals mediated by the IgA surface BCR on plasma cells affect their localization, survival, and function? IgA plasmablasts and, more importantly, mature IgA plasma cells still express IgA-BCRs on their cell surface (77, 214–216). However, the biological function of their IgA-BCR expression remains elusive. One could envision that IgA-BCRs on plasma cells initiate tonic signals similar to the BCR on B cells (217). IgA-BCR signals might also be triggered by antigen and continuous or repetitive antigen-binding might be crucial for survival and/or inducing regulatory functions of plasma cells, such as cytokine production. For IgM plasma cells it was demonstrated that antigen binding to the surface IgM-BCR resulted in transcriptional changes and altered cytokine profiles (218). Signals mediated *via* the IgA-BCR might also contribute to the localization of IgA plasma cells within the villus.

Hence, new genetic models in combination with ST need to be developed to investigate the biological relevance of the surface IgA-BCR and its signals in the context of IgA plasma cell function and longevity.

## Author contributions

KP and WS conceptualized and wrote the manuscript. JW, FK, and H-MJ revised the manuscript. All authors contributed to the article and approved the submitted version.
